# Plectasin: from evolution to truncation, expression, and better druggability

**DOI:** 10.3389/fmicb.2023.1304825

**Published:** 2023-12-21

**Authors:** Xuan Li, Ya Hao, Na Yang, Ruoyu Mao, Da Teng, Jianhua Wang

**Affiliations:** ^1^Gene Engineering Laboratory, Feed Research Institute, Chinese Academy of Agricultural Sciences, Beijing, China; ^2^Innovative Team of Antimicrobial Peptides and Alternatives to Antibiotics, Feed Research Institute, Chinese Academy of Agricultural Sciences, Beijing, China; ^3^Key Laboratory of Feed Biotechnology, Chinese Academy of Agricultural Sciences, Department of Agriculture and Rural Affairs, Beijing, China

**Keywords:** plectasin, evolution, truncation, expression, druggability

## Abstract

Non-computational classical evolution analysis of plectasin and its functional relatives can especially contribute tool value during access to meet requirements for their better druggability in clinical use. *Staphylococcus aureus* is a zoonotic pathogen that can infect the skin, blood, and other tissues of humans and animals. The impact of pathogens on humans is exacerbated by the crisis of drug resistance caused by the misuse of antibiotics. In this study, we analyzed the evolution of anti-*Staphylococcus* target functional sequences, designed a series of plectasin derivatives by truncation, and recombinantly expressed them in *Pichia pastoris* X-33, from which the best recombinant Ple-AB was selected for the druggability study. The amount of total protein reached 2.9 g/L following 120 h of high-density expression in a 5-L fermenter. Ple-AB was found to have good bactericidal activity against gram-positive bacteria, with minimum inhibitory concentration (MIC) values ranging between 2 and 16 μg/mL. It showed good stability and maintained its bactericidal activity during high temperatures, strong acid and alkali environments. Notably, Ple-AB exhibited better druggability, including excellent trypsin resistance, and still possessed approximately 50% of its initial activity following exposure to simulated intestinal fluids for 1 h. *In vitro* safety testing of Ple-AB revealed low hemolytic activity against mouse erythrocytes and cytotoxicity against murine-derived macrophages. This study successfully realized the high expression of a new antimicrobial peptide (AMP), Ple-AB, in *P. pastoris* and the establishment of its oral administration as an additive form with high trypsin resistance; the study also revealed its antibacterial properties, indicating that truncation design is a valuable tool for improving druggability and that the candidate Ple-AB may be a novel promising antimicrobial agent.

## Introduction

*Staphylococcus aureus* (*S. aureus*) is a gram-positive bacterium that frequently attacks the skin, soft tissues, and bloodstream of humans, colonizing many parts of the body and causing host infections (Pollitt et al., 2018; Pidwill et al., 2020; Mohammad et al., 2021). Atopic dermatitis caused by *S. aureus* is one of the most common skin diseases in children, affecting up to a quarter of children in the UK and Ireland (Harkins et al., 2018). Meanwhile, *S. aureus* bloodstream infections are one of the most common and severe bacterial infections worldwide; they caused a high risk of metastatic complications of up to 20–30% and many in-hospital mortalities (Kim et al., 2004; Jung and Rieg, 2018). The fundamental reason for the prevalence of *S. aureus* is that it has developed resistance to almost all the classes of antibiotics, and infections caused by drug-resistant strains of *S. aureus* have reached global epidemic proportions. The overall burden of staphylococcal diseases, particularly those caused by methicillin-resistant strains of *S. aureus* (MRSA), is increasing in healthcare and community settings in many countries (Assis et al., 2017). The discovery of penicillin ushered in the ‘age of antibiotics,’ but it took less than a decade for penicillin resistance to emerge (Chambers and DeLeo, 2009). The use of antibiotics such as streptogramin, virginiamycin, florfenicol, and rifampicin over the last few decades has not solved the problem of antibiotic resistance but has rather exacerbated this crisis (Foster, 2017). More worryingly, resistance to cell membrane-targeting daptomycin and cell wall-targeting vancomycin, the last line of antibiotics against *S. aureus*, has successively emerged (Jahan et al., 2022; Yang et al., 2023). The first case of vancomycin-resistant *Staphylococcus aureus* was reported in Japan in 1997, followed by sporadic reports of daptomycin- and vancomycin-resistant strains (Humphries et al., 2013; Miller et al., 2016). Although these resistant strains have not spread frequently on a large scale, they are still a strong warning that the search for new antibacterial drugs is imperative.

As important components of the host innate defense system, antimicrobial peptides (AMPs) have a narrower window of mutation-selection and a more diverse mechanism of action than other classic antimicrobial drugs, thereby reducing the risk of bacterial resistance development (Chen et al., 2018; Jiang et al., 2021; Li et al., 2021). Simultaneously, the high intracellular activity of AMPs and their good synergy with other antibiotics results in a low residual level, low dosage, and longer administration interval of the drug in organisms (Yang et al., 2021, 2023). In another aspect, AMPs are from innate defensing system with natural compatibility in body playing as a role of peace keeper during daily health maintenance unlike the traditional antibiotics and chemical drugs specially for overcoming intensive pathogens and severe infections usually with the obvious side effects as residual, cytotoxic, resistance and others via role of heavy weapons or killers; In fact, the former is usually more important than the latter as a whole on purpose of green health, and they are connected each other closely via a balance keeping attributing into the same goal, it is of unique great importance especially under the almost dry or empty pipeline of new drug R & D for years; Corresponding, the iron triangle theory of the health protection built by AMPs, vaccines, and antibiotics is conducive to maintaining a reasonable balance among pathogens, antimicrobial agents, and drug-resistance (Hao et al., 2022). However, AMPs also have some drawbacks, such as low stability due to enzyme sensitivity, high toxicity, and low bioavailability (Wang et al., 2021; Aminov, 2022). The instability of AMPs is a major impediment to their entry into clinical studies, especially the enzymatic sensitivity of peptides that are extremely susceptible to degradation by proteases in the gastrointestinal (GI) tract and tissue proteolysis when injected, which limits their oral and intravenous availability. To date, only one peptide drug has been approved for clinical use as a GLP-1 receptor antagonist and is permitted for oral use, semaglutide (Wang et al., 2022). Therefore, it is essential to obtain a peptide drug that is highly stable and resistant to protease hydrolysis in the blood, tissue, brain and GI environments. Numerous studies have shown that the biological characteristics of AMPs are closely related to their physicochemical properties such as their positive charge, amphiphilicity, and secondary structure (Yang et al., 2017; Bonnel et al., 2020). The advantages of derivative peptides, such as arenicin-3, 17KKV, and others demonstrate the feasibility of the molecular design of a peptide by deleting or replacing some amino acid residues to change its physicochemical properties and obtain a more efficient and stable antimicrobial peptide (Elliott et al., 2020; Takada et al., 2022). However, the question of how to delete or substitute has been suspended without a clear answer; currently, the only feasible way is to randomly try in different laboratories, which requires a huge industrial labor input.

**Figure 1:**
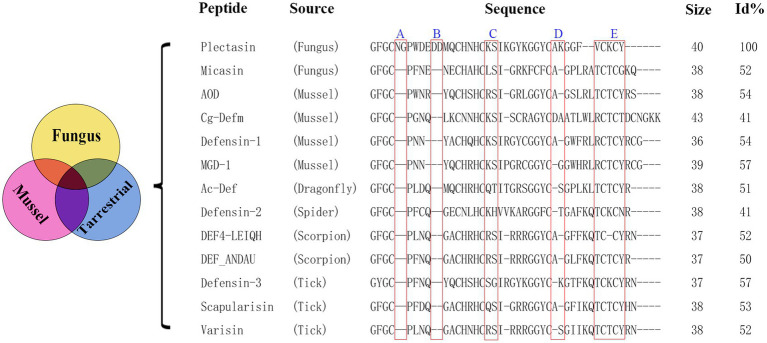
Multiple sequence alignment of plectasin and some antibacterial ancient invertebrate-type defensins (AITDs). Size is the number of amino acids. Sequence identity (%) to plectasin is shown on the right [Id (%) stands for sequence identity.]. The Id (%) was obtained by protein BLAST (Basic Local Alignment Search Tool) in NCBI (National Center for Biotechnology Information). Read boxes indicate the 5 selected truncated fragments. Sequence information references Mygind et al. (2005), Wilmes and Sahl (2014), and Wu et al. (2014).

Plectasin was the first fungal defensin isolated from *Pseudoplectania nigrella* (Mygind et al., 2005) and has been taken as a typical model of similar fungal metabolites for nearly 20 years since its discovery in 2005. It borrows the interaction with the peptidoglycan precursor, Lipid II, to inhibit the synthesis of the cell wall for bactericidal purposes (Schneider et al., 2010; Liu et al., 2020; Pohl et al., 2022). Plectasin has antibacterial activity against gram-positive bacteria, especially *Streptococcus* and *Staphylococcus* (Brinch et al., 2009), however, it is trypsin-sensitive and highly cytotoxic at high concentrations (Zhang et al., 2011; Yang et al., 2019). Although the derivative peptides of plectasin, NZ2114 and MP1106, show stronger antimicrobial activity to some extent due to their high CPP (cell-penetrating peptide) activity as an additional source force, the shortcomings of trypsin sensitivity and higher cytotoxicity have not been well addressed (Wang et al., 2018). Here, using a non-computer-aided classical experiential BI (bioinformatics) tool, we performed multiple sequence alignment analyses between plectasin and 12 other antibacterial ancient invertebrate-type defensins (AITDs) with a similar conserved cysteine-stabilized α-helix and β-sheet structural (CSαβ) motif and similar antimicrobial activity based on previous reports (Figure 1; Mygind et al., 2005; Mandal et al., 2009; Wilmes and Sahl, 2014; Wu et al., 2014) to find an operation entrance for further improvement. In this study, we compared the effect of five different oligopeptide fragments (three types of oligopeptide regions, including redundant, easy mutation/indetermination, and C-terminal) with plectasin via DNA truncating. These five fragments were named as followed: A: Asn5-Gly6, B: Asp11-Asp12, C: Lys20-Ser21, D: Ala30-Lys31, and E: Val36-Cys37-Lys38-Cys39-Tyr40. Ple-AB lacking four amino acids (Asn5, Gly6, Asp11, and Asp12) was selected as the only candidate with bioactivity, and this was confirmed by yeast expression and activity screening. We achieved efficient expression of the antimicrobial peptide Ple-AB in *P. pastoris* X-33, deduced its structure–function relationship, characterized its druggability and evaluated its ability to combat *S. aureus in vitro*.

## Materials and methods

### Peptide design

Since plectasin was discovered in 2005 (Mygind et al., 2005), other fungal defensin-like peptides (fDLPs) have been also identified from a diverse species by a comparative genomics approach, On the basis of sequence, structural, and functional similarity, these defensins can be divided into some major types (Wu et al., 2014), plectasin is classified as antibacterial ancient invertebrate-type defensins (AITDs). We selected sequences that share the conserved sequence of plectasin which is antibacterial activity specially and among different species that lived in different environments, and delete possible redundant fragments (A and B) at the same time. By comparison, we find their similarities and differences, meanwhile, we hope to design antimicrobial fragments that can be used in a wide range of environments, including forests, oceans and others. At present, synthetic biology, chemical biosynthesis, and artificial intelligence still are difficult to accurately reconstruct the original appearance and details of biological evolution over hundreds of millions of years, and our experiential molecular design is therefore worthy of expenditure, experimentation and expectation.

A series of plectasin derivatives were designed based on multiple sequence alignments. The ExPASy server was used to analyze differences in the physicochemical properties between the derived and parent peptides.

### Expression and purification

The recombinant expression vector was constructed by ligating the designed and optimized sequence to the pPICZαA inducible expression vector, relying on the preference of the codon of *P. pastoris*. The linearized plasmid was transferred into *P. pastoris* X-33 for expression and the resulting product was verified for activity using an inhibition zone assay (against *S. aureus* ATCC 43300) before the active peptide was expressed at a high density in a 5-L fermenter (Mao et al., 2015). The supernatant was purified using the AKTA Express system. The fermentation supernatant was collected by centrifugation (5,000 rmp, 30 min), filtered with micro-filtration membranes (0.45 mm). According to the isoelectric point of the target product, the corresponding A (50 mM sodium phosphate buffer, pH 6.7) and B (50 mM sodium phosphate buffer, 600 mM NaCl, pH 6.7) buffers were configured and purified by HiPrep SP FF cation exchange column (GE Healthcare). The protein samples were eluted by increasing the NaCl concentration in a stepwise manner, and the corresponding elution peak was collected. The purified product was verified using tricine-sodium dodecyl sulfate-polyacrylamide gel electrophoresis (Tricine-SDS-PAGE) and matrix-assisted laser desorption/ionization–time-of-flight mass spectrometry (MALDITOF MS) (Zhang et al., 2014).

### Structure analysis

The secondary structure of the AMPs was analyzed by circular dichroism (CD). The Ple-AB was quantified to 70 μg/mL using ddH_2_O, 20 mM sodium dodecyl sulfate (SDS), and 50% trifluoroethanol (TFE), to simulate hydrophobic environments and microbial membranes (Liu et al., 2020). The detailed operation is outlined in a previous experiment (Yang et al., 2019).

### Antibacterial activity of Ple-AB

#### Minimum inhibitory concentration

The minimum inhibitory concentration (MIC) of the peptides was assayed using the micro broth dilution method, which is an important indicator of the antibacterial activity of the AMPs (Liu et al., 2021). MIC plates were prepared by adding 10 μL of peptide solution (different concentrations of 10–1,280 μg/mL at a 2-fold ratio) and 90 μL of bacterial suspension (1 × 10^5^ CFU/mL, logarithmic growth stage) to a 96-well microtiter plate; and the same amount (10 μL) of sterile saline was used as a negative control. The MIC was defined as the lowest concentration of ones at which no visible bacterial growth was observed. All the assays were repeated thrice.

#### Bacterial kinetics assay *in vitro*

The bactericidal efficiency of Ple-AB was evaluated by plotting a time-kill curve (Zheng et al., 2021). Single colonies of the *S. aureus* ATCC43300 were picked for overnight activation, and 1% were transferred and incubated at 37°C until the logarithmic growth phase. The bacterial broth (1 × 10^5^ CFU/mL) was incubated at 37°C and 250 rpm with final concentrations of 1×, 2×, and 4× MIC of AMPs and antibiotics. Samples of 100 uL of bacteria were taken at 0, 0.5, 1, 2, 4, 6, 8, 10, 12, and 24 h, diluted, and coated to count the number of single colonies and plot the time-killing curve.

#### Post-antibiotic effect

*Staphylococcus aureus* ATCC43300 in the logarithmic growth phase was diluted to 1 × 10^8^ CFU/mL with fresh culture medium, incubated with 1×, 2×, and 4× MIC Ple-AB at 37°C for 2 h, and then diluted 1,000 times with MHB to terminate the bactericidal effect of the AMPs or antibiotics. They were incubated at 37°C and 250 rpm, sampled, and coated on MHA plates for colony counting every hour. Phosphate-buffered saline (PBS) was used as a negative control, and vancomycin was used as a positive control. The calculation formula for the Post-antibiotic effect (PAE) was based on previous study (Wu et al., 2022).

### Biosafety and stability of Ple-AB

#### Hemolysis

The hemolytic properties of the peptides were evaluated by measuring the amount of hemoglobin released from fresh mouse erythrocytes (Yang et al., 2019). Blood was collected from mice in sodium heparin anticoagulation tubes, and the erythrocytes were washed three or more times with 0.9% saline to obtain an 8% erythrocyte suspension. The erythrocyte suspension was mixed with an equal volume of Ple-AB at concentrations of 0.5–256 μg/mL, incubated at 37°C, and the absorbance values were measured at 540 nm. PBS and 0.1% Triton X-100 were used as the blank (A_0_) and positive controls (A_100_), respectively. Hemolysis (%) = [(A – A_0_)/(A_100_ – A_0_)] × 100, and three replicates were conducted for each case.

#### Cytotoxicity

The cytotoxicity of Ple-AB toward the murine macrophage RAW 264.7 (Peking Union Medical College) was tested using the MTT assay (Wang et al., 2018). Cells (2.5 × 10^4^ CFU/mL) were incubated at 37°C for 24 h, and thereafter Ple-AB at a concentration of 2–256 μg/mL was added for 24 h. The supernatant was washed twice, 5 μg/mL 3-(4,5-dimethylthiazol-2-yl)-2,5-diphenyltetrazolium bromide (MTT) solution was added, and the mixture was incubated for 4 h. The absorbance of each well was measured at 570 nm using a microplate reader. PBS (A_control_) was used as the control. Survival rate (%) = (A/A_control_) × 100 was calculated.

#### *In vivo* safety

Eight 6-week-old ICR mice were randomly divided into two groups and injected intraperitoneally with Ple-AB (10 mg/kg, body weight) and saline for six consecutive days. The mice were observed for mobility, feeding, and other behaviors. They were weighed daily, and blood was collected on the 7th day for hematological and biochemical analyses. Simultaneously, the heart, liver, spleen, lungs, and kidneys were collected for tissue fixation and hematoxylin–eosin (HE) staining to observe any pathological changes (Shishkina et al., 2023).

#### Thermal, pH, salts, serum, and protease stability

The stability of Ple-AB was assessed based on its antibacterial activity against *S. aureus* ATCC43300. Temperature stability was determined by incubation at 20, 40, 60, 80, and 100°C for 1 h (Li et al., 2020). The pH stability was determined by incubation in buffers with a pH of 2, 4, 6, 8, and 10 for 4 h (Yang et al., 2017). The peptides were incubated with different concentrations of metal ions, serum, artificial gastric fluid, and simulated intestinal fluid to assess their stability in the blood and intestines (Lyu et al., 2019). All the treatments were performed in triplicates.

## Results

### Peptide design based on a classical non-computational bioinformatic tool

Based on plectasin and its special antibacterial activity, the sequences and their alignment analysis of 13 similar defensins from publications are shown in Figure 1; these were derived from six different species (fungus, mussel, dragonfly, spider, scorpion, and tick). Plectasin shares 41–57% sequence similarity with 12 other similar defensins from fungi and invertebrates. In comparison with other AITDs, plectasin had additional Asn5, Gly6 (fragment A), and anionic amino acids (Asp11-Asp12, fragment B) in its N-loop region, suggesting that A and B do not contribute into antimicrobial activity. Their original role in native livings source is unknown, and a similar situation existed in fragments C and D (Figure 1; Wilmes and Sahl, 2014; Wu et al., 2014, 2022). In this work, we tried the classical non-computational DNA editing technique instead of modern CRISPR editing for the macro-genome, focusing on the improvement/reconstruction of the complex metabolism and non-synthetic biology using a modern computer-aided design that focuses on the fine *de novo* design for core drug candidates. Firstly, five key conserved residues were kept unchangeable except for special purposes (Mygind et al., 2005). Next, 15 new peptides were designed by simple DNA truncating to obtain three types of oligopeptide regions, including redundancy (A, B), easy mutation/indetermination (C, D) and the C-terminal region (E) of plectasin. Except for region E, we analyzed and considered the former four fragments as useless for antibacterial activities due to their weak or unclear bioinformation regarding antibacterial activity contribution and support. Because 12 other peptides from different species were completely lacking regions A and B (Figure 1), these two were selected first for truncation. Similarly, C and D were regarded as easy mutation or non-constituent regions for special antibacterial functions. Finally, the last fragment E, which contains two key resides Cys-37 and Cys-39, was deleted to check whether it is necessary for structure and function as a whole. Therefore, in this experiment, we selected the five target oligopeptide fragments and sequentially truncated them individually or in combination to construct 15 new peptide derivatives of plectasin, hoping to achieve better derivatives that merge the merits of druggability. The five new peptides Ple-A, Ple-B, Ple-C, Ple-D and Ple-E were constructed by individually truncating A, B, C, D, and E, respectively. Moreover, another six new peptides, Ple-AB, Ple-AC, Ple-AD, Ple-BC, Ple-BD, and Ple-CD were constructed by truncating six two-combination fragments, respectively; and more four new peptides Ple-ABC, Ple-ABD, Ple-ACD and Ple-BCD were constructed by truncating four three-combination fragments, respectively, as a scaffolding and free climbing tool (Table 1).

**Table 1:** Physicochemical properties of peptides derived from plectasin.

No.	**Name**	**Sequence**	**Length**	**M.W.**	**PI**	**Charge**	**GRAVY**	**II**
0	Plectasin	GFGCNGPWDEDDMQCHNHCKSIKGYKGGYCAKGGFVCKCY	40	4407.99	7.77	+1	−0.695	13.82
1	Ple-A	GFGCPWDEDDMQCHNHCKSIKGYKGGYCAKGGFVCKCY	38	4236.83	7.77	+1	−0.629	23.05
2	Ple-B	GFGCNGPWDEMQCHNHCKSIKGYKGGYCAKGGFVCKCY	38	4177.81	8.62	+3	−0.547	8.96
3	Ple-C	GFGCNGPWDEDDMQCHNHCIKGYKGGYCAKGGFVCKCY	38	4192.74	6.87	0	−0.608	14.02
4	Ple-D	GFGCNGPWDEDDMQCHNHCKSIKGYKGGYCGGFVCKCY	38	4208.74	6.87	0	−0.676	17.89
5	Ple-E	GFGCNGPWDEDDMQCHNHCKSIKGYKGGYCAKGGF	35	3811.23	6.88	0	−0.909	14.37
6	Ple-AB	GFGCPWDEMQCHNHCKSIKGYKGGYCAKGGFVCKCY	36	4006.66	8.62	+3	−0.469	18.42
7	Ple-AC	GFGCPWDEDDMQCHNHCIKGYKGGYCAKGGFVCKCY	36	4021.58	6.87	0	−0.533	23.77
8	Ple-AD	GFGCPWDEDDMQCHNHCKSIKGYKGGYCGGFVCKCY	36	4027.58	6.87	0	−0.606	26.13
9	Ple-BC	GFGCNGPWDEMQCHNHCIKGYKGGYCAKGGFVCKCY	36	3962.56	8.30	+2	−0.447	8.90
10	Ple-BD	GFGCNGPWDEMQCHNHCKSIKGYKGGYCGGFVCKCY	36	3978.56	8.30	+2	−0.519	11.26
11	Ple-CD	GFGCNGPWDEDDMQCHNHCIKGYKGGYCGGFVCKCY	36	3993.48	6.00	−1	−0.583	16.61
12	Ple-ABC	GFGCPWDEMQCHNHCIKGYKGGYCAKGGFVCKCY	34	3791.40	8.30	+2	−0.359	18.92
13	Ple-ABD	GFGCPWDEMQCHNHCKSIKGYKGGYCGGFVCKCY	34	3807.40	8.30	+2	−0.435	21.41
14	Ple-ACD	GFGCPWDEDDMQCHNHCIKGYKGGYCGGFVCKCY	34	3822.33	6.00	−1	−0.503	27.08
15	Ple-BCD	GFGCNGPWDEMQCHNHCIKGYKGGYCGGFVCKCY	34	3763.31	7.78	+1	−0.412	11.33

Length: amino acid number; MW, molecular weight (Da); PI, isoelectric point; GRAVY, grand average of hydropathicity; II, instability index; http://web.expasy.org/protparam/.

### Bioinformatics analysis of the derived peptides from truncation design

The sequences of the 15 novel peptides from plectasin truncation were analyzed for their physicochemical properties using biological software. These new derived peptides were constructed by composing of 34–38 amino acids lacking five different fragments, with an isoelectric point range of 6.00–8.62, a positive charge number of −1 ~ +3, and increased hydrophobicity compared to plectasin (Table 1), from our experiences of the recombination expression of many defensin AMPs (Zhang et al., 2011, 2014; Li et al., 2017; Yang et al., 2019; Liu et al., 2020; Hao et al., 2023; Jin et al., 2023). We cannot exactly estimate and know their possible expression results but only try them one by one, even though they have shown good feasibility of recombination expression based on their net charge number, pI, instability index, and other parameters, and also we cannot know whether they have normal biological activities expect testing.

### Construction of recombinant plasmids and screening for positive transformants

**Figure 2:**
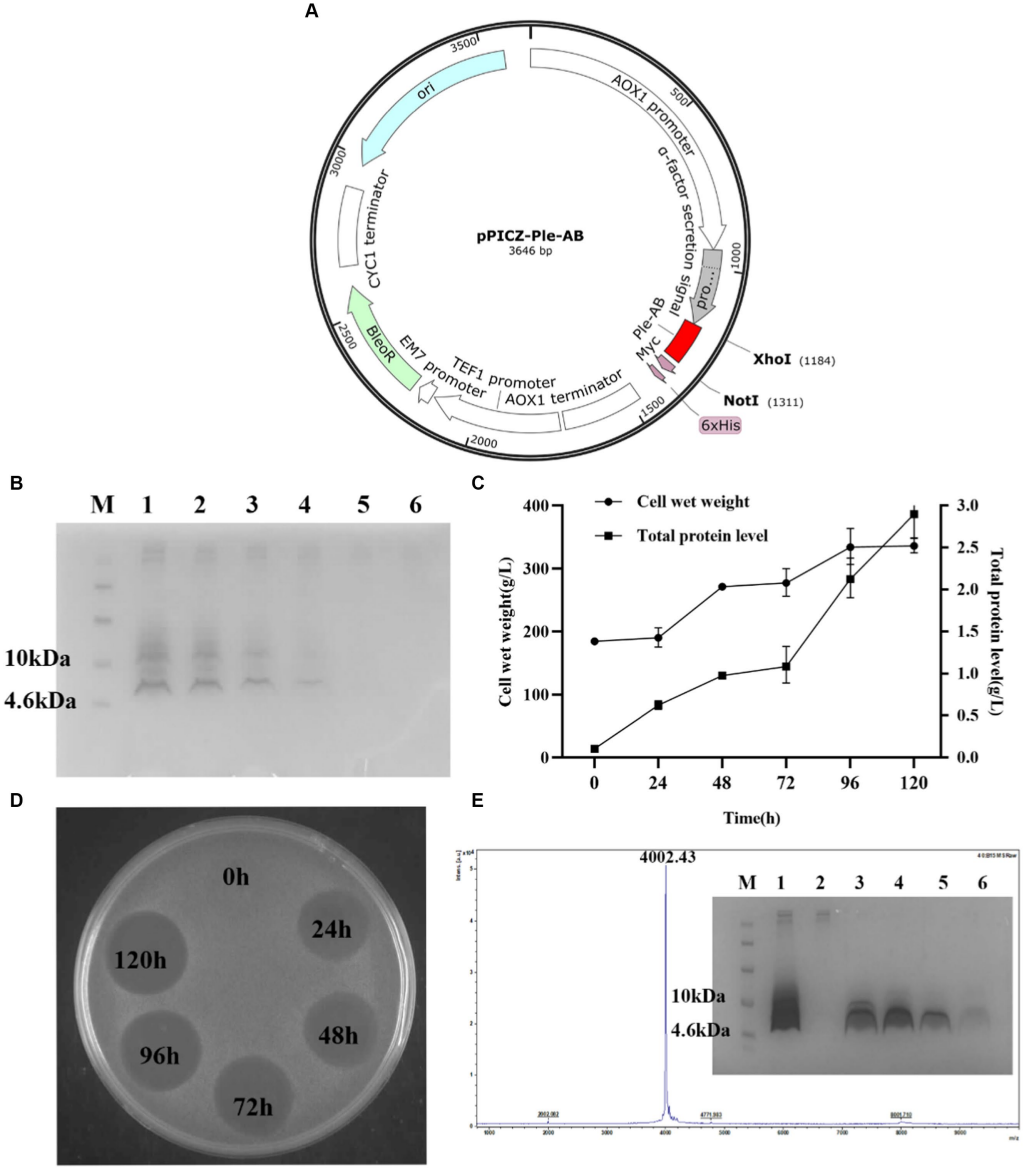
Expression and purification of Ple-AB in *P. pastoris* X-33 at 5 L fermentor. **(A)** Schematic representation of the recombinant expression plasmid pPIC-Ple-AB. **(B)** Tricine-SDS PAGE gel electrophoresis for Ple-AB expression. Lane M indicated the protein molecular weight marker (6 μL). Lane 1–6 indicated fermentation supernatants of Ple-AB (10 μL) taken at 120, 96, 72, 48, 24, 0 h of induction, respectively. **(C)** Time curves of Ple-AB total secreted protein levels and cell wet weight during high density fermentation. **(D)** Inhibition of *S. aureus* ATCC43300 by Ple-AB fermentation supernatant with different induction times. **(E)** MALDI-TOF MS analysis of purified Ple-AB and Tricine-SDS-PAGE analysis of Ple-AB purified by cation exchange chromatography. Lane 1: 120 h fermentation supernatant; Lane 2: penetration peak; Lanes 3–4: 22.5% B-eluting peak; Lane 5-6: 60% B-eluting target peak.

A total of 15 recombinant plasmids were constructed. As shown in Figure 2A (using Ple-AB as an example), following double digestion by *Xho*I and *Not*I, the codon-optimized sequences of the Ple-A–Ple-BCD gene were inserted into the pPICZαA plasmid. The 15 types of recombinant plasmids pPIC-Ple-A, …, pPIC-Ple-BCD were linearized using *Pme*I and transferred into *P. pastoris* X-33 receptor cells. The positive transformants (96 for each sequence) were selected and cultured in 48-well plates for 120 h. The induced supernatant of the transformants was analyzed by inhibition circle screening and Tricine-SDS-PAGE, and the results indicated that Ple-AB exhibited better antibacterial activity. Four Ple-AB-positive transformants with good inhibitory effects were selected for expression in 1-L shake flasks using the same method. The expression levels were 203, 176, 174, and 137 mg/L, and the transformants with the highest expression were selected for the high-density induced expression in a 5-L fermenter.

### Expression and purification

Transformants of Ple-AB with the correct sequence and high expression were selected for the high-density expression in 5-L fermenters. After 120 h of induced expression, the total secreted protein and total biomass of Ple-AB were 2.90 g/L and 332.5 g/L, respectively, and the target band of Ple-AB (4 kDa) was clearly observed in the gel. It is clear from the inhibition circle diagram that antibacterial activity increased with fermentation time (Figures 2B–D). Subsequently, purification was performed using cation-exchange chromatography, and the corresponding elution peak of Ple-AB was observed and collected. As shown in Figure 2E, a single target band was obtained by Tricine-SDS-PAGE, and the relative molecular weight of the purified protein was verified to be 4002.43 Da using MALDI-TOF MS, which is consistent with the theoretical relative molecular mass of Ple-AB (4006.66 Da).

### Structure analysis

**Figure 3:**
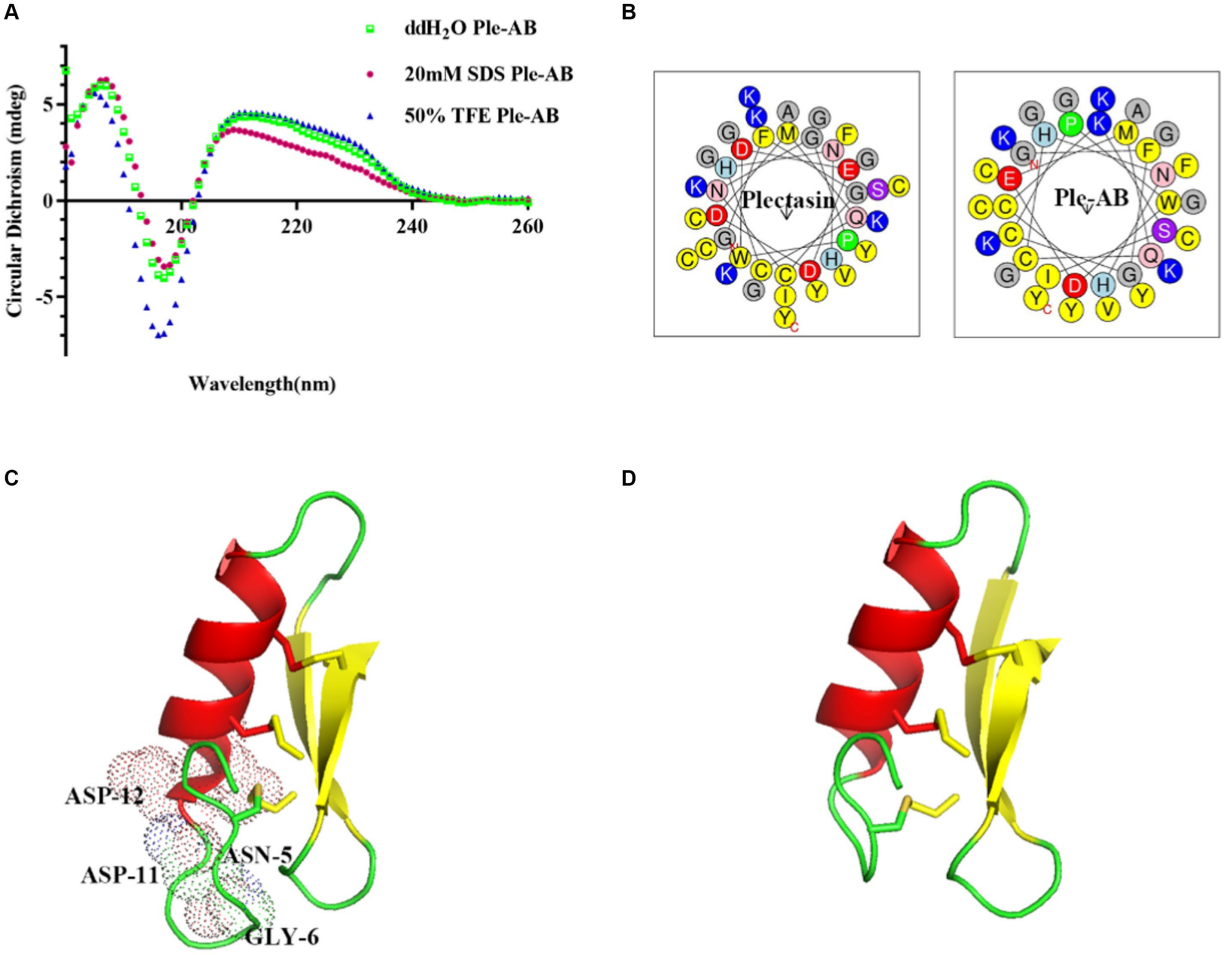
The structure analysis of plectasin and Ple-AB. **(A)** CD spectra of Ple-AB in ddH_2_O, 20 mM SDS, and 50% TFE. **(B)** Observations on the amphipathy of plectasin and Ple-AB in the spiral wheel model (yellow represents hydrophobic amino acids, red is negatively charged acidic amino acids, blue is positively charged basic amino acids and purple is hydrophilic amino acids). **(C)** Molecular mimicry of plectasin. Light dots represent truncated residues. **(D)** Molecular mimicry of Ple-AB. Molecular model was generated with PyMOL 1.8.

The secondary structure of Ple-AB was analyzed using CD. The results showed that Ple-AB had positive peaks at 186 nm and 211 nm and a negative peak at 197 nm. In addition, the α-helices of Ple-AB increased in SDS; this alteration may facilitate the entry of Ple-AB into the microbial cell membrane. Meanwhile, it was found that the random coil of Ple-AB increased in the TFE solution, inferring that Ple-AB may not easily cause damage to the mammalian cell membrane (Figure 3A and Table 2). The amphipathy of plectasin and Ple-AB is observed in the helical wheel diagram in Figure 3B; compared to plectasin, Ple-AB has two additional positive charges and increased hydrophobicity. The results of the 3D structural simulations predicted that Ple-AB is structurally similar to plectasin with a typical CSαβ conformation (Mandal et al., 2009), including an α-helix (residues 8–16), an antiparallel β-sheet layer (residues 23–27 and 32–36) and three disulfide bonds (Cys4-Cys26, Cys11-Cys33 and Cys15-Cys35) (Figures 3C,D).

**Table 2:** Ratio of secondary structure of Ple-AB in different solutions.

Indices (%)	ddH_2_O	20 mM SDS	50% TFE
Helix	3.08	3.57	2.53
Antiparallel	55.60	56.45	43.58
Parallel	3.17	3.29	2.73
Beta-turn	13.25	13.08	15.98
Random coil	25.09	23.60	35.09

### Antimicrobial activity of Ple-AB

#### Minimum inhibitory concentration determination

The antibacterial spectrum of Ple-AB is shown in Table 3. The results showed that Ple-AB exhibited good antibacterial activity against Gram-positive bacteria, especially against *Streptococcus* including *S. agalactiae* ATCC13813, *S. dysgalactiae* CVCC3938, and *S. agalactiae* CAU-FRI-2022-02, with MICs in the range of 2–4 μg/mL. Ple-AB was also found to be inactive against gram-negative bacteria. Meanwhile, the MIC of Ple-AB against *Candida albicans* CICC 98001 was determined, as shown in Table 3, which was greater than 128 μg/mL.

**Table 3:** The MIC values of Ple-AB.

Strains	MIC (μg/mL)	MIC (μg/mL)	MBC (μg/mL)	Source
	Plectasin	Ple-AB		
Gram-positive bacteria				
*S. aureus* ATCC43300	4^a^	16	32	ATCC
*S. aureus* ATCC25923	NT	16	64	ATCC
*S. aureus* CVCC546	16^a^	8	16	CVCC
*S. aureus* E48	4^a^	4	16	NWAFU
*S. epidermidis* ATCC35984	16	4	8	ATCC
*S. epidermidis* ATCC12228	8	2	8	ATCC
*S. dysgalactiae* CVCC3938	NT	4	4	CVCC
*S. agalactiae* ATCC13813	2	4	4	ATCC
*S. agalactiae* CAU-FRI-2022-01	NT	4	32	CAU
*S. agalactiae* CAU-FRI-2022-02	NT	2	4	CAU
Gram-negative bacteria				
*E. coli* ATCC25922	>128	>128	NT	ATCC
*E. coli* O157	>128 ^a^	>128	NT	CVCC
*Shigella* CMCC3926	>128	>128	NT	CGCC
*S. enteriditis* CVCC3377	>128	>128	NT	CVCC
*P. aeruginsoa* CICC21630	>128 ^a^	>128	NT	CICC
Fungus				
*Candida albicans CICC 98001*	>128	>128	NT	CICC

ATCC, American Typical Culture Collection; CVCC, China Veterinary Microbial Strain Collection Management Centre; CICC, China Center of Industrial Culture Collection; CMCC, National Center for Medical Culture Collection. The bovine clinical *S. aureus* E48 strain was donated by Prof. Zhao Xin (Northwest A&F University). *S. agalactia* CAU-FRI-2022-01 and CAU-FRI-2022-02 isolated from bovine mastitis tissue were obtained from China Agricultural University (CAU). NT, no test. ^a^Data were cited from previous results (Yang et al., 2019).

#### *In vitro* bacterial kinetics assay

**Figure 4:**
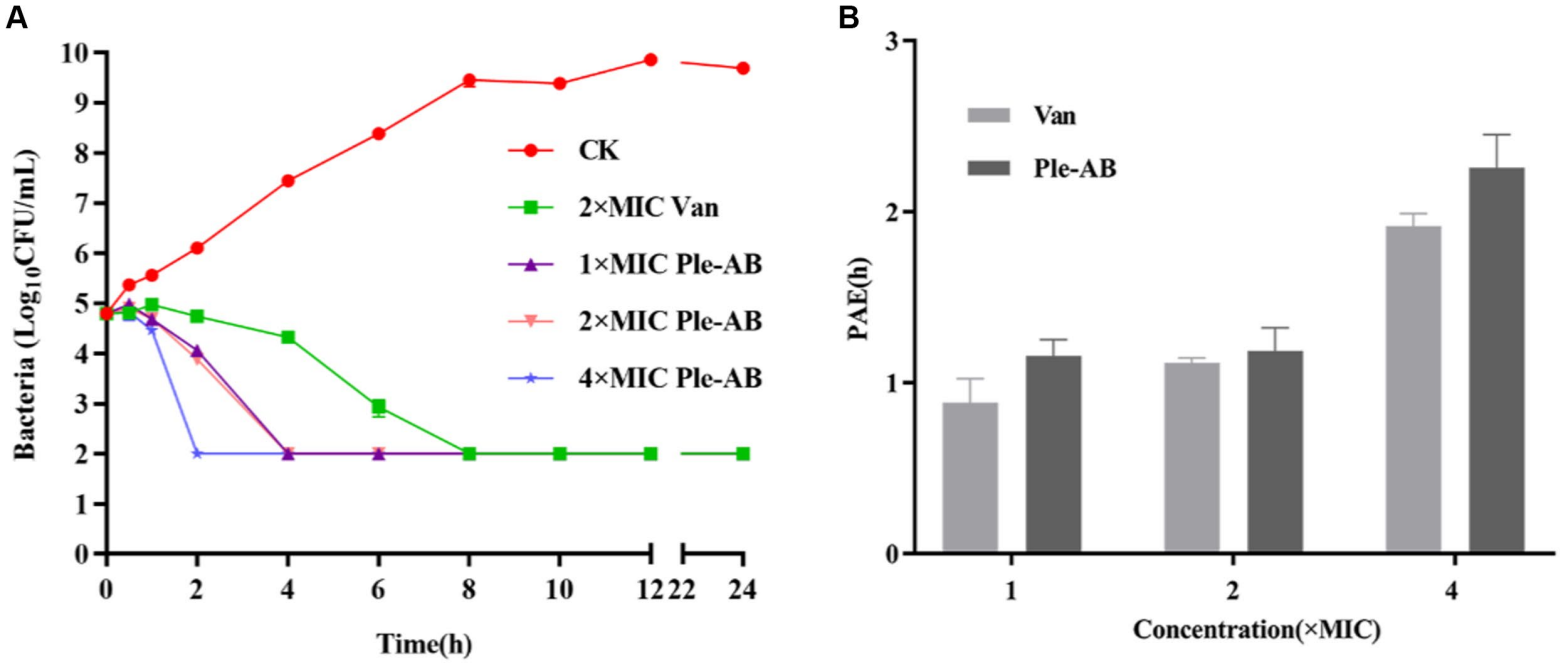
Bactericidal profile and post-antibiotic effect of Ple-AB. **(A)** Time killing curves of 1×, 2×, and 4× MICs of Ple-AB against *S. aureus* ATCC43300. **(B)** Post-antibiotic effect of 1×, 2×, and 4× MIC of Ple-AB against *S. aureus* ATCC43300. Van stands for the vancomycin.

*Staphylococcus aureus* ATCC43300 was used as the test strain and the bactericidal ability of Ple-AB was examined based on the kinetics of bactericidal time (Figure 4A). The 1× and 2× MIC Ple-AB were able to kill all the strains within 4 h, and the 4× MIC Ple-AB achieved 99% bactericidal activity within 2 h. However, the 2× MIC vancomycin (the MIC value of vancomycin for *S. aureus* ATCC43300 was 1 μg/mL) required 6 h to kill all the strains, which was weaker than that of Ple-AB.

#### Post-antibiotic effect

The post-antibiotic effect is an important parameter in evaluating the pharmacokinetics of new drugs. The PAE was determined by treating *S. aureus* ATCC43300 with 1×, 2×, and 4× MIC antimicrobial agents for 2 h (Figure 4B). The PAE values of Ple-AB for *S. aureus* ATCC43300 were dose-dependent and exhibited better inhibition than vancomycin at 1×, 2×, and 4× MIC concentrations for the 1.15, 1.18, and 2.25 h time points, respectively.

### Ple-AB had low toxicity, and high stability

#### Low toxicity

**Figure 5:**
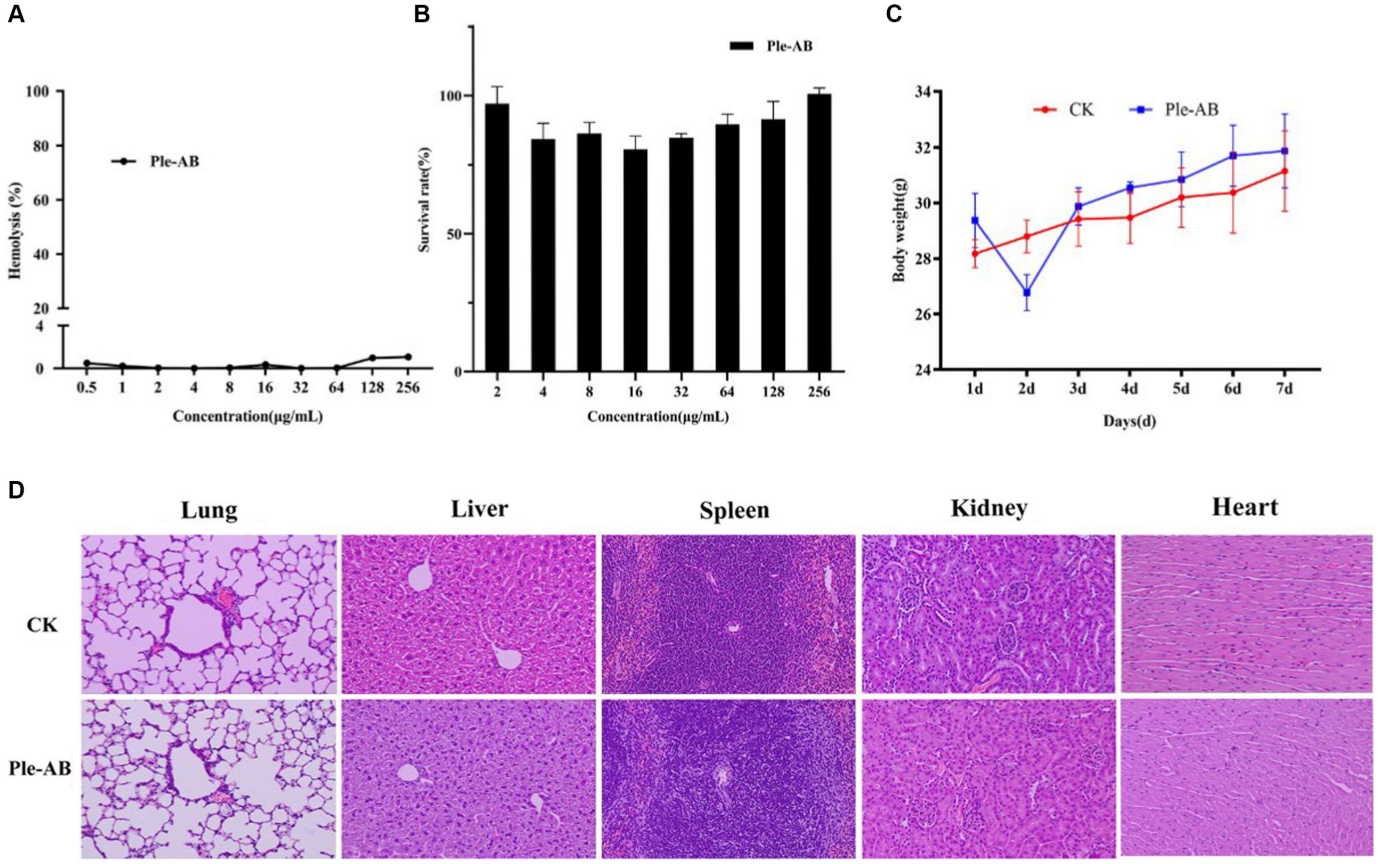
Safety of Ple-AB. **(A)** Hemolytic activity of Ple-AB at different concentrations (0.5–256 μg/mL) against mouse erythrocytes. **(B)** Cytotoxic effects of Ple-AB at different concentrations (2–256 μg/mL) on mouse macrophages RAW264.7. **(C)** Body weight changes and **(D)** tissue sections by HE (magnification 200×) of mice after injection of Ple-AB 1 week.

To evaluate safety, the hemolytic activity and cytotoxicity of Ple-AB were determined. The hemolysis of mouse erythrocytes treated with different concentrations (0.5–256 μg/mL) of Ple-AB was first analyzed, and the results showed that the hemolytic rates were all less than 2%, even at the highest concentration, the hemolytic rate was only 1.07% (Figure 5A). The cytotoxicity of Ple-AB was then measured using a MTT assay, and the results showed that there was no significant cytotoxicity to the mouse peritoneal macrophages RAW264.7 at concentrations of 2–256 μg/mL, and the cell survival rate was above 80% (Figure 5B). This indicated that Ple-AB has a good *in vitro* safety profile.

#### *In vivo* safety

During the safety test cycle in mice, it was observed that the mice had a slight tendency to lose weight on the second day after the injection; however, on the third day, the weight of the mice returned to normal (Figure 5C). One week after the intraperitoneal injection, HE staining of the heart, liver, spleen, lungs, and kidneys showed no differences in histological morphology (Figure 5D). The whole blood cell profiles and serum biochemical indices were not significantly different between the test and control group (Tables 4–6).

**Table 4:** Hematological indices in mice.

Parameters	WBC (10^9^/L)	NEUT (10^9^/L)	LYM (10^9^/L)	MONO (10^9^/L)	EO (10^9^/L)	BASO (10^9^/L)	NEUT %	LYM %	MONO %	EO %	BASO %
CK	7.30 ± 1.72	1.28 ± 0.392	5.50 ± 1.161	0.20 ± 0.045	0.23 ± 0.113	0.08 ± 0.026	17.42 ± 1.85	75.56 ± 3.15	2.72 ± 0.57	3.19 ± 0.85	1.12 ± 0.21
Ple-AB	7.89 ± 0.94	1.49 ± 0.354	5.89 ± 0.585	0.24 ± 0.066	0.19 ± 0.106	0.09 ± 0.027	18.78 ± 1.66	74.8 ± 3.02	3 ± 0.49	2.33 ± 1.24	1.09 ± 0.34

**Table 5:** Hematological indices in mice.

Parameters	RBC (10^12^/L)	HBG (g/L)	HCT %	MCV (fL)	MCH (pg)	MCHC (g/L)	PLT (10^9^/L)	MPV (fL)	PDW %	PLCR %	RDW-CV (10^9^/L)	RDW-SD %	PCT %
CK	9.75 ± 0.5	129.25 ± 9.25	40.63 ± 2.17	40.08 ± 1.52	12.88 ± 0.57	32.81 ± 1.03	775.5 ± 53.5	6.53 ± 0.67	15.4 ± 1.1	13.95 ± 1.25	19.38 ± 0.72	42.95 ± 3.35	0.02 ± 0.006
Ple-AB	9.44 ± 0.82	128 ± 12	41.3 ± 2.4	41.73 ± 1.17	13.48 ± 0.67	32.86 ± 1.73	782.3 ± 48.25	6.63 ± 0.57	15.85 ± 0.75	15.35 ± 3.65	18.58 ± 1.97	44.55 ± 3.65	0.02 ± 0.01

**Table 6:** Serum biochemical indices in mice.

Parameters	Glucose (mmol/L)	Ca^2+^ (mmol/L)	Phosphorous (mmol/L)	Urea Nitrogen (mmol/L)	Creatinine (μmol/L)	Total protein (g/L)	Albumin (g/L)	Globulin (g/L)	ALB/GL0	AST (IU/L)	ALT (IU/L)	AST/ALT
CK	1.60 ± 0.14	1.39 ± 0.05	3.24 ± 0.29	9.4 ± 0.58	85.57 ± 6.03	53.81 ± 3.54	34.98 ± 2.53	18.83 ± 0.50	1.86 ± 0.12	25.72 ± 1.56	47.15 ± 4.82	0.55 ± 0.06
Ple-AB	1.59 ± 0.15	1.38 ± 0.06	3.33 ± 0.20	11.6 ± 0.8	83.13 ± 4.57	54.41 ± 4.56	35.59 ± 4.58	18.82 ± 0.25	1.90 ± 0.25	25.85 ± 2.33	49.07 ± 6.21	0.53 ± 1.11

#### High stability

*Staphylococcus aureus* ATCC43300 was used as the test strain to analyze the *in vitro* stability of Ple-AB (Table 7). Ple-AB was incubated at different temperature and pH values ranging from 20 to 100°C and 2–10, respectively; the MIC values increased twofold only at 100°C and pH 10, but were not affected under the other conditions. After incubating Ple-AB with different concentrations of serum and ions, the antibacterial activity remained unchanged, indicating that Ple-AB is highly stable (Table 7).

**Table 7:** Thermal, pH, salts, and protease stability of Ple-AB against *S. aureus* ATCC 43300.

Treatment		MIC (μg/mL)
		Ple-AB
	Control	16
Temperature		
	20°C	16
	40°C	16
	60°C	16
	80°C	16
	100°C	32
pH		
	2	16
	4	16
	6	16
	8	16
	10	32
Salts		
	Na^+^ (150 mM)	16
	K^+^ (4.5 mM)	16
	NH_4_^+^ (6 μM)	16
	Zn^2+^ (8 μM)	16
	Mg^2+^ (1 mM)	16
	Ca^2+^ (2.5 mM)	16
	Fe^3+^ (4 μM)	16
Simulated Gastric Fluid (1-2 h)		16
Artificial Intestinal Fluid (1 h)		32
Serum (25%) (1–8 h)		16
Serum (50%) (1–8 h)		16

Ple-AB was incubated with artificial gastric juice for 2 h, and the MIC value remained at 16 μg/mL, indicating that it is tolerant to pepsin. It is worth noting that Ple-AB still possessed approximately 50% of its initial activity following incubation in the simulated intestinal fluids for 1 h and lasted for 2 h of incubation, indicating that Ple-AB is tolerant to trypsin (Table 7). Therefore, Ple-AB exhibited desirable stability; this is vital for GI administration and has the importance of applications, as only one case different from other fungi defensins and the derivatives has been reported so far.

## Discussion

*Staphylococcus aureus* is the most common pathogen in septic infections, causing localized septicemia, pneumonia, pericarditis, pseudomembranous enteritis and, and in severe cases, systemic infections such as septicemia and sepsis (Paling et al., 2017; Lei et al., 2018; Pollitt et al., 2018). *S. aureus* can cause persistent and recurrent infections and lead to the constant emergence of drug-resistant strains, posing a serious threat to human, animal, and public health safety (Guo et al., 2020). In this study, truncation, a simple DNA editing operation, was used as a powerful tool to improve the druggability of the fungal defensin plectasin; moreover, Ple-AB showed the strong antimicrobial activity against *Staphylococcus* at low MIC values of 4–16 μg/mL (Table 3).

Many efforts have been made by researchers to bring plectasin, an extremely promising fungal defensin, to the early stages of clinical application (Brinch et al., 2009). In 2011, our group expressed and purified recombinant plectasin in *P. pastoris* with a total secretion of 0.748 g/L following 120 h of induction in recombinant yeast (Zhang et al., 2011). Various peptide derivatives have been designed to increase expression; most of these derivative peptides were designed and constructed based on the mutation or substitution of single or multiple amino acids, which is the most common and efficient method (e.g., NZ2114, MP1102) (Andes et al., 2009; Zhang et al., 2015; Koseki et al., 2023). These mutants showed significantly increased expression of *P. pastoris* and exhibited high antibacterial activity.

However, their poor druggability remains a challenge. AMPs are derived from a wide variety of plants, animals, and microorganisms, and vary highly with environmental factors such as temperature, humidity, and other factors; they live in different locations, resulting in their sequence diversity across species. Based on previous reports, we selected 12 defensins from six species with sequences, structures and functions similar to that of plectasin for the multi-sequence alignment (Mygind et al., 2005; Wilmes and Sahl, 2014; Wu et al., 2014). It is known that the antimicrobial action of plectasin depends on the five key amino acids Phe2, Gly3, Cys4, His18, and Cys37, in which, the Phe2, Gly3, Cys4, and Cys37 interact with the pyrophosphate group of the bacterial cell-wall precursor Lipid II via hydrogen bonding, in addition, and a salt bridge is formed between the side chain of His18 and the D-γ-glutamic acid of Lipid II, thus inhibiting cell-wall biosynthesis (Mandal et al., 2009; Schneider et al., 2010). Other peptides also contain the five key amino acids involved in the plectasin-Lipid II interaction, signifying a conserved lipid II-binding motif (Figure 1). A truncation instead of a substitution approach was used to improve the properties of plectasin for the first time in this study. Five fragments, A–E (Figure 1) were selected for truncation. In comparison with the other 12 defensins, only plectasin has two spare redundant fragments, A and B, in its N-loop region (Figure 1). We examined whether the deletion of fragments A and B affect the activity of the Plectasin. The C-segment is a mutation-prone region at the end of the *α*-helical structure. The D-fragment is an indetermination region in the *β*-sheet parallel structure, and the E-fragment function as a control and is relatively conserved in a sheet at the C-terminal. Simultaneously, these three fragments were selected for deletion to study their effect. Therefore, the above core residues, groups, and domains remined unchanged, except for the E-terminus, during our truncation operation. Screening revealed that none of the truncated segments individually showed activity. Interestingly, only Ple-AB, following the simultaneous truncation of fragments A and B, showed normal or higher activity; the other combined truncated derived peptides showed no activity. This mean that A and B have an equal role as structural or functional supports; this is reported for the first time in our study. Of greater importance is that fragments A and B seem no contribution to the apparent antibacterial function, but are essential as the plectasin source living, i.e., in *Pseudoplectania nigrella* (Mygind et al., 2005) as a whole in their environment. Moreover, the fact that double truncation of AB improved its expression and druggability especially in antitrypsin proteolysis reminds that not only fragment AB might play roles as the balancing of symmetrical structure in peptide, co-handshaking and co-locking during targeting toward pathogens, but also as the possible special trypsin sensitive region, they both are probably key sites that host trypsin access, recognize and bind specifically with plectasin. However, to reveal further details about how important fragments A and B from *Pseudoplectania nigrella* (Mygind et al., 2005) are to other fungi and invertebrates would be very interesting but is beyond the aim or scope of our study. Similar details regarding fragments C and D are worth studying in subsequent steps.

The length, positive charge number, and amphiphilicity of the derived peptide Ple-AB were altered, reducing the peptide chain length from 40 to 36 amino acids, reducing the molecular weight from 4407.99 to 4006.66 Da, increasing the charge number from +1 to +3, and increasing the hydrophobicity from −0.695 to −0.469 (Table 1). The high-density expression of Ple-AB also increased, with a total secretion of 2.9 g/L following 120 h of induction in recombinant yeast (Figure 2C), while the expression level of plectasin was relatively low 748.63 mg/L (Zhang et al., 2011); the cytotoxicity (<1%) and hemolytic (1.07%) activity were also significantly reduced (Figure 5B) compared to plectasin (<5.31 and 68%, respectively) at the concentration of 256 μg/mL (Zhang et al., 2011; Yang et al., 2019). Ple-AB also exhibited superior antibacterial effects against vancomycin in terms of its bactericidal kinetics and PAE *in vitro* (Figures 4A,B). Notably, its stability *in vitro* was the above characteristics of Ple-AB were significantly better than that of the other peptide derivatives, with better druggability contributing directly into its application steps and progress.

It is worth noting that plectasin and its series of derivative peptides lost activity after 15–30 min of co-incubation with the simulated intestinal fluid (Lei et al., 2018), but the tolerance or resistance of Ple-AB to trypsin was highly ameliorated, still possessing approximately 50% of its initial activity after incubation with the simulated intestinal fluids for 1 h (Table 7). In the same simulated *in vitro* environment, the trypsin tolerance time of Ple-AB was three times greater than that of plectasin and its series of derivative peptides. Clinical applications require drugs that exhibit high structural stability in complex and variable *in vivo* environments. During oral administration, many factors, including solubility, absorption, and stability, affect the oral bioavailability of a drug. The most challenging factor for the gastrointestinal transport of therapeutic proteins is proteolysis, (Loussouarn et al., 2020) which restricts golden time and stability ensuring its feasibility during application. Protein hydrolases are classified into two main groups, exopeptidases and endopeptidases. To resist the exopeptidases, they can often be blocked by “end-capping.” This is a chemical modification of the N-terminal and C-terminal amino or carboxylic acid functional groups (Brinckerhoff et al., 1999). For trypsin as an endopeptidase, common resistance improvements include sequence modification, cyclization, incorporation of unnatural amino acids, introduction of peptidomimetic elements, and nanoengineering of antimicrobial peptide polymers (Choi et al., 2013; Koopmans et al., 2015; Zhao et al., 2016). These strategies attempt to modify or mask the potential peptide cleavage sites as much as possible to reduce protease recognition and proteolysis, with the side effect of increasing uncertainty in the prediction of biological activity (Zhu et al., 2020). In general, the shorter the peptide, the stronger the proteolysis resistance. This is because shorter molecular lengths may result in fewer total cleavage sites and greater access difficulty for various enzymes, including tryptic degradation. Thus, reducing the size of the peptide is also a viable strategy to resist trypsin (Zou et al., 2022). The deletion of four amino acids in Ple-AB compared to plectasin not only resulted in a reduction in the overall length, but the truncated amino acids also likely changed the amphiphilicity of the whole peptide structure (Figure 3B), thus contributing to better druggability. The optimization of amphiphilicity can improve protein stability (Stone et al., 2019); the stability results of Ple-AB confirm the possibility of this deduction, provides a new idea and tool for improving trypsin resistance, and is an important step toward the early availability of cationic antimicrobial peptides as orally active drugs.

As we know, both CRISPRR/Cas and computer-aided design are powerful intelligent tools, particularly for the complex macro-biomolecular characteristics in higher living organisms and drug candidates from synthetic chemicals. However, they are not suitable for the design, modification, and preparation of *de novo* defensins and other AMPs with strong cationic polarity and low stability. Surprisingly, simple DNA truncation guided by evolution analysis proved to be a powerful free climbing and scaffolding tool to successfully try and improve druggability in this work. Its importance of methodology could be highly expected from plectasin as the first defensin and model AMP in fungi with better feasibilities and possibilities during screening for new antibacterial agents from fungi source.

Overall, this work reminds us that truncation is an effective and powerful tool to carry out simple DNA editing for the purpose of improving defensin druggability, and its feasibility has been confirmed in this study. The successful high-density expression of Ple-AB in *P. Pastoris* contributes to a reduction in the cost of peptide drugs, and the better antibacterial characteristics specifically for druggability and safety shown by Ple-AB *in vivo* and *in vitro* also prove it to be a better drug candidate. The reason that Ple-AB increases tolerance to trypsin and its antimicrobial mechanism still need to be explored and disclosed.

## Data availability statement

The datasets presented in this study can be found in online repositories. The names of the repository/repositories and accession number(s) can be found in the article/supplementary material.

## Ethics statement

The animal study was approved by the Animal Care and Use Committee of the Feed Research Institute of Chinese Academy of Agricultural Sciences. The study was conducted in accordance with the local legislation and institutional requirements.

## Author contributions

XL: Data curation, Formal analysis, Investigation, Writing – original draft, Methodology. YH: Funding acquisition, Methodology, Validation, Writing – review & editing, Resources, Conceptualization, Supervision. NY: Methodology, Validation, Writing – review & editing. RM: Methodology, Resources, Writing – review & editing. DT: Methodology, Resources, Writing – review & editing, Project administration. JW: Conceptualization, Funding acquisition, Project administration, Resources, Validation, Writing – review & editing, Supervision, Methodology.
